# Supplementary figures and images for: Blue Light Exposure Caused Large-Scale Transcriptional Changes in the Abdomen and Reduced the Reproductive Fitness of the Fall Armyworm Spodoptera frugiperda
Source: Insects. 2023 Dec 26;15(1):10. doi: 10.3390/insects15010010 (PMC10816951; doi:10.3390/insects15010010)

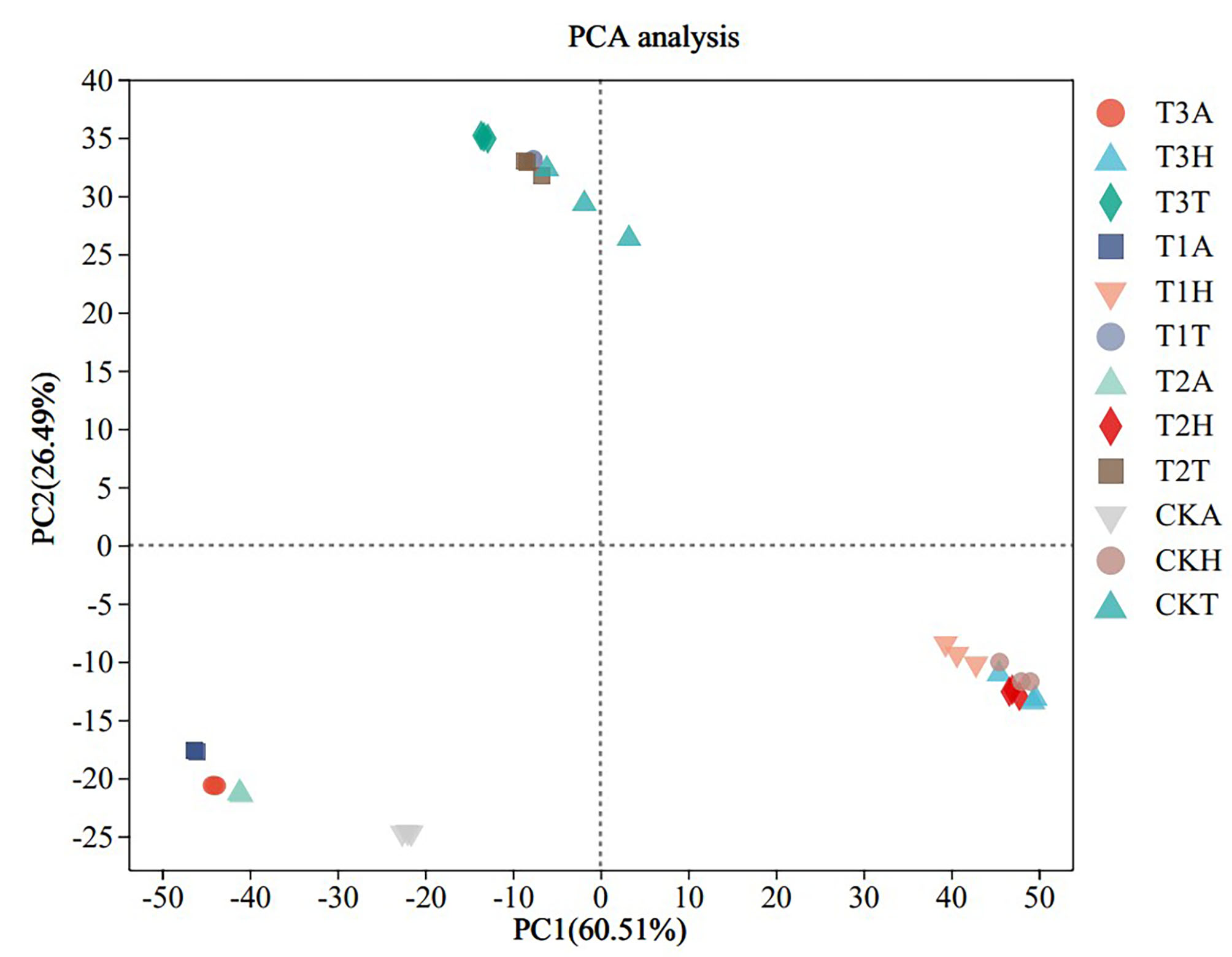

Supplement: Supplementary file 1 [file insects-15-00010-s001.zip › Fig. S1.tif]

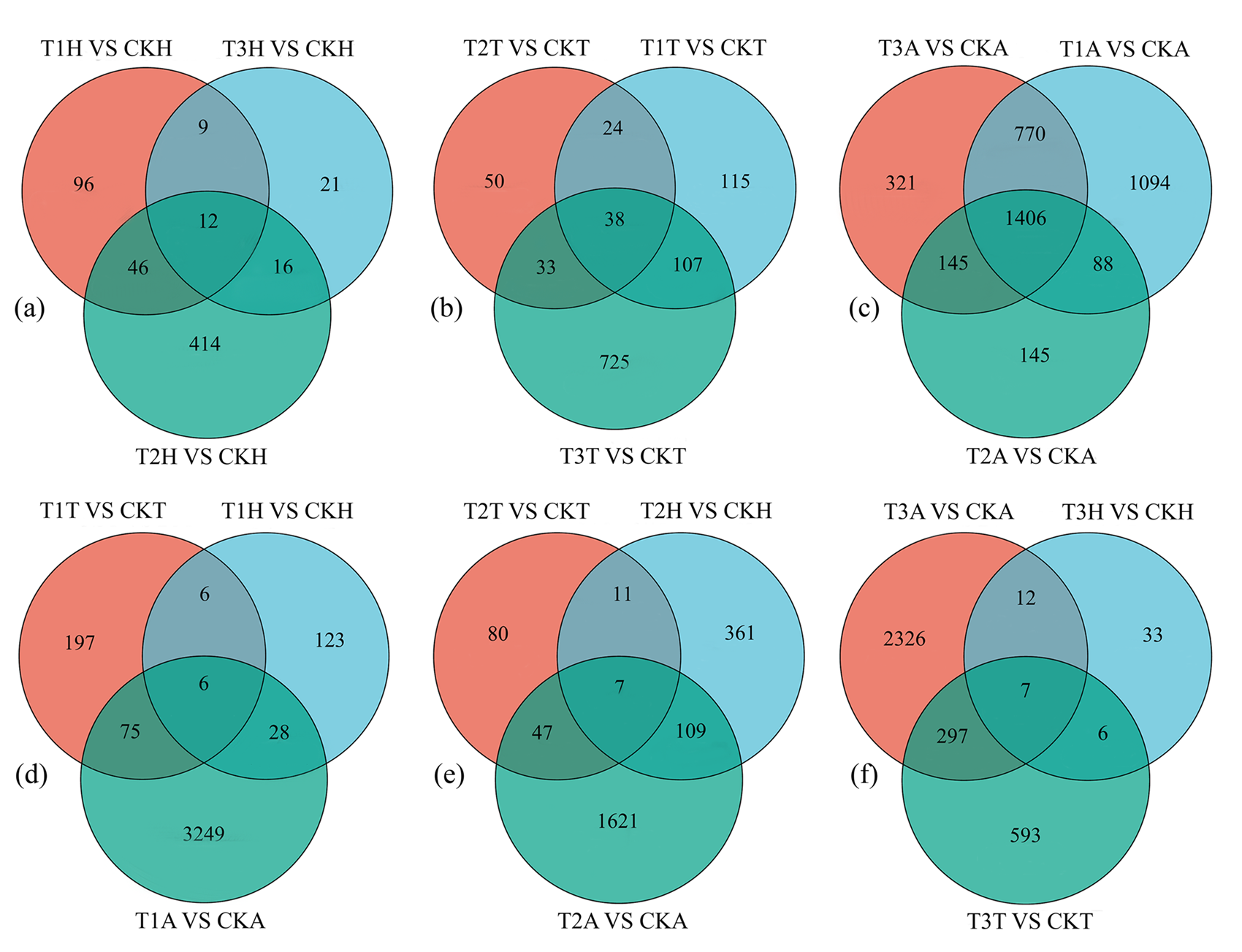

Supplement: Supplementary file 1 [file insects-15-00010-s001.zip › Fig. S2.tif]

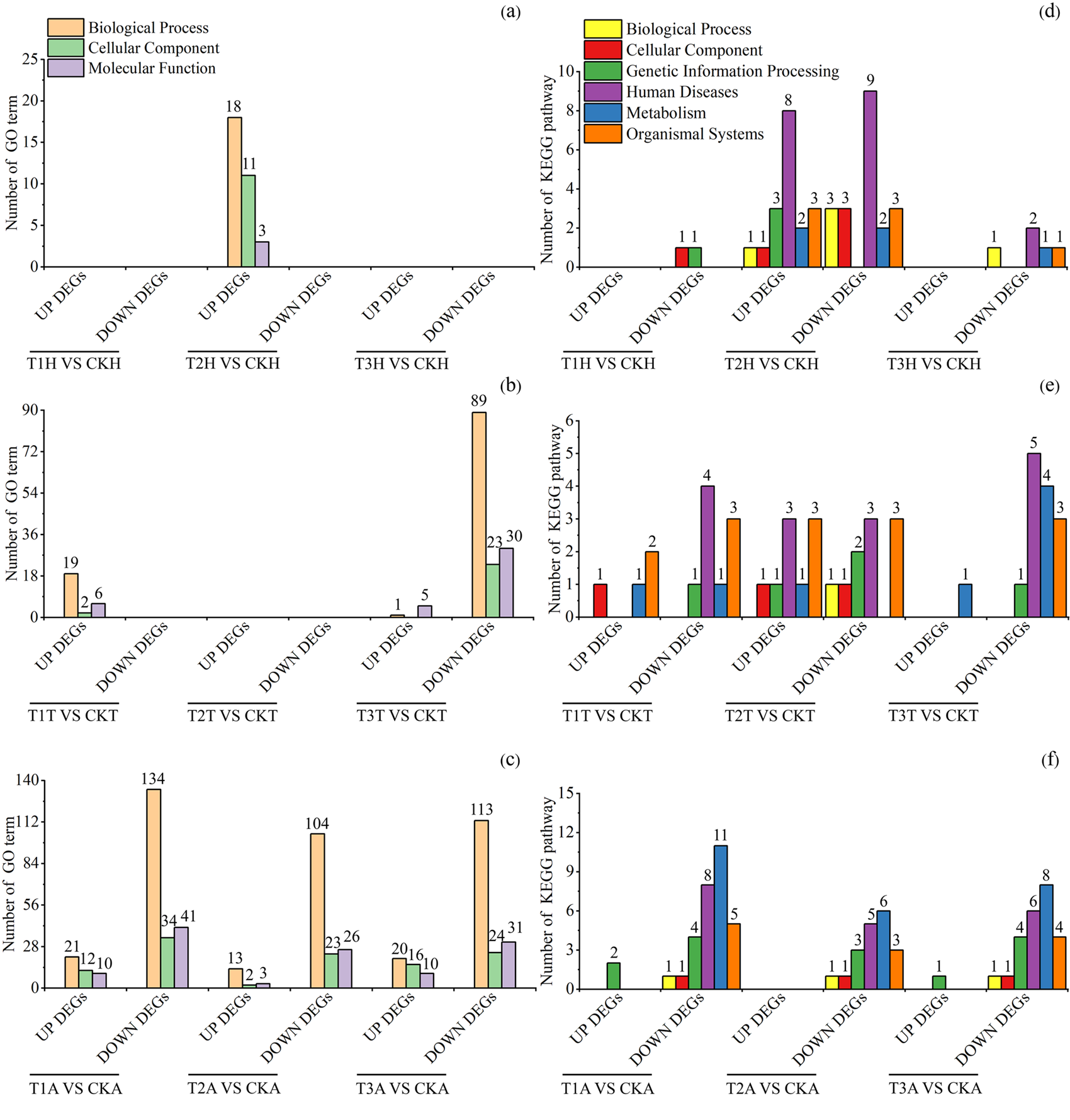

Supplement: Supplementary file 1 [file insects-15-00010-s001.zip › Fig. S3.tif]

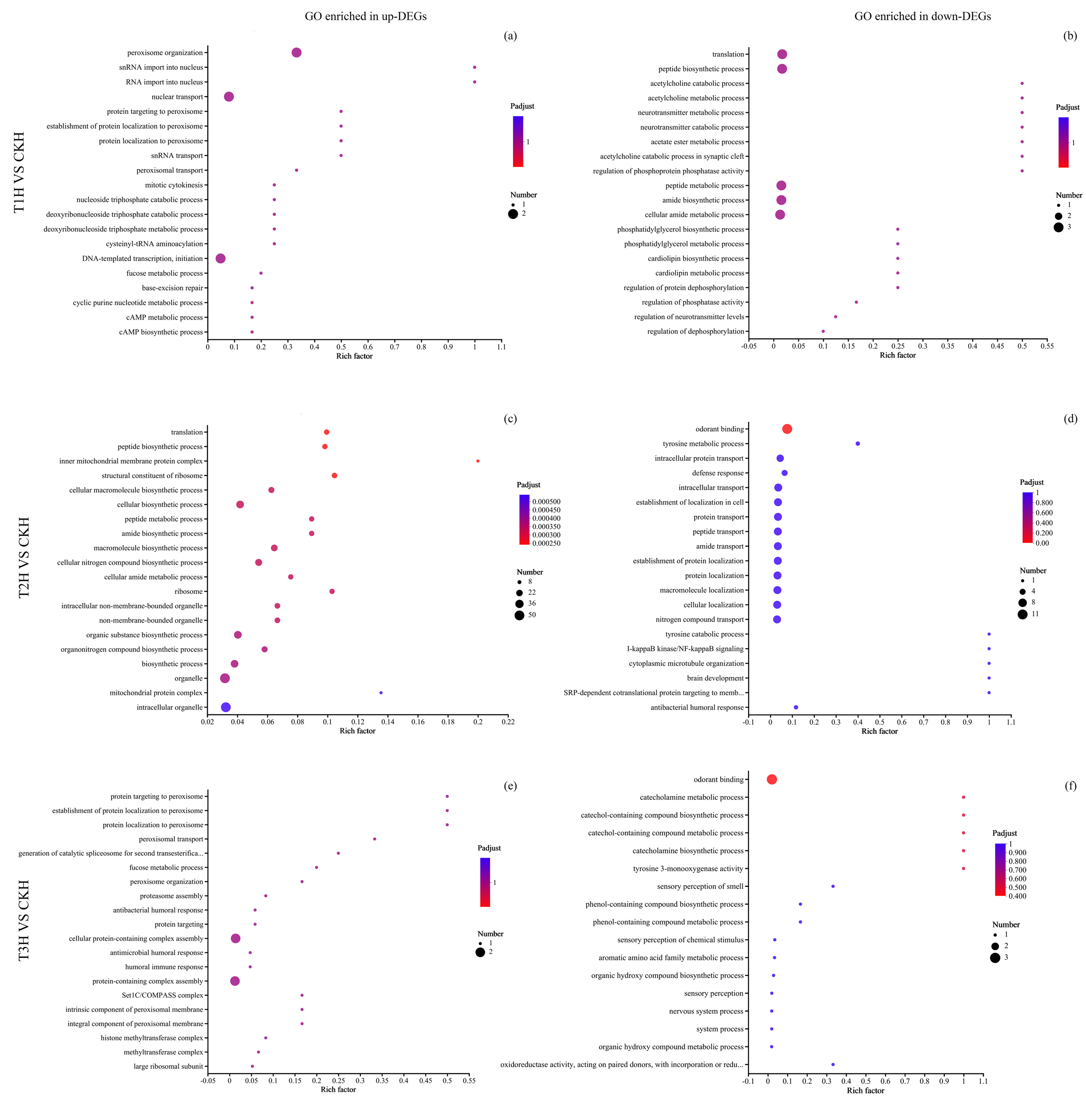

Supplement: Supplementary file 1 [file insects-15-00010-s001.zip › Fig. S4.tif]

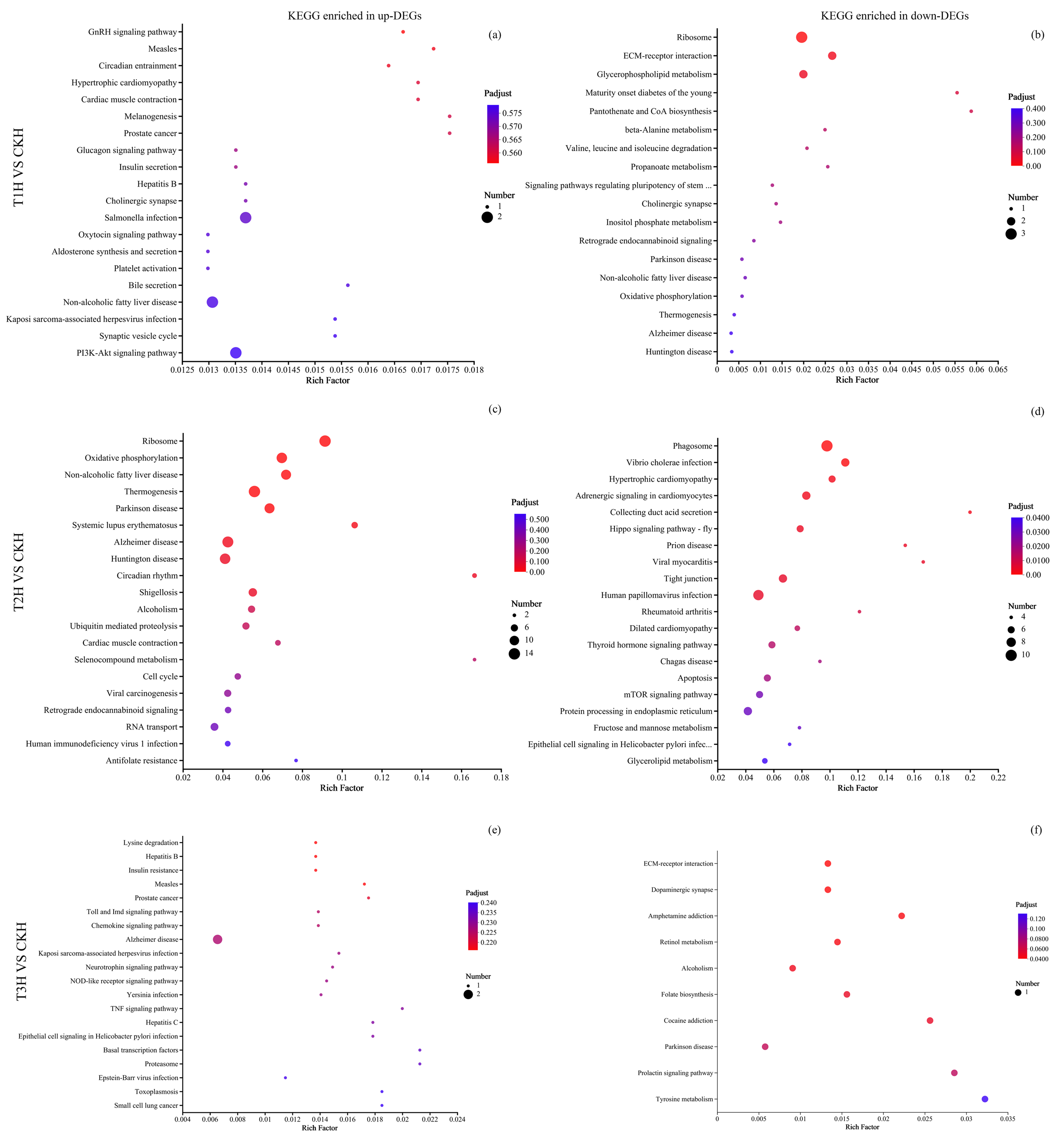

Supplement: Supplementary file 1 [file insects-15-00010-s001.zip › Fig. S5.tif]

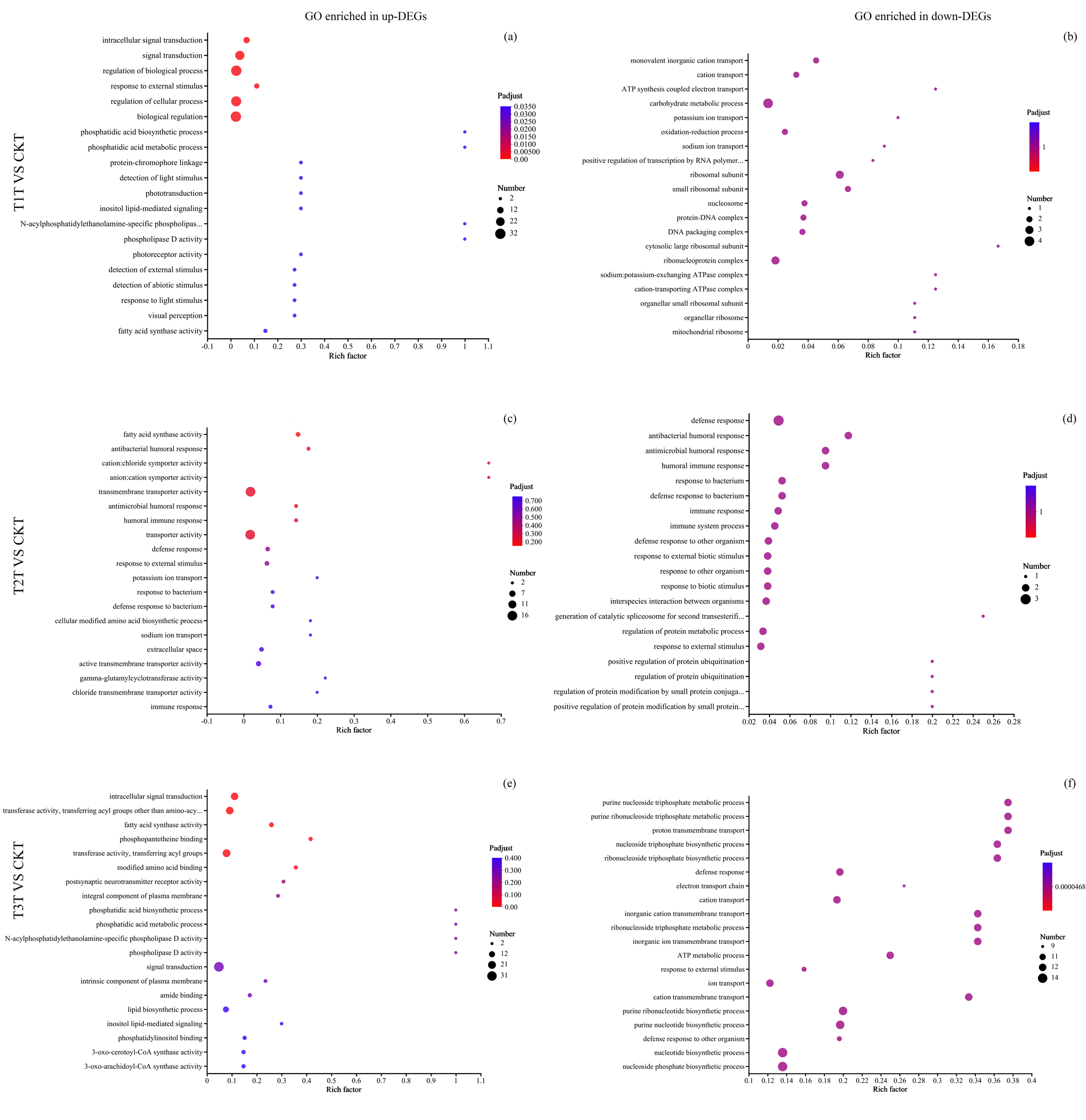

Supplement: Supplementary file 1 [file insects-15-00010-s001.zip › Fig. S6.tif]

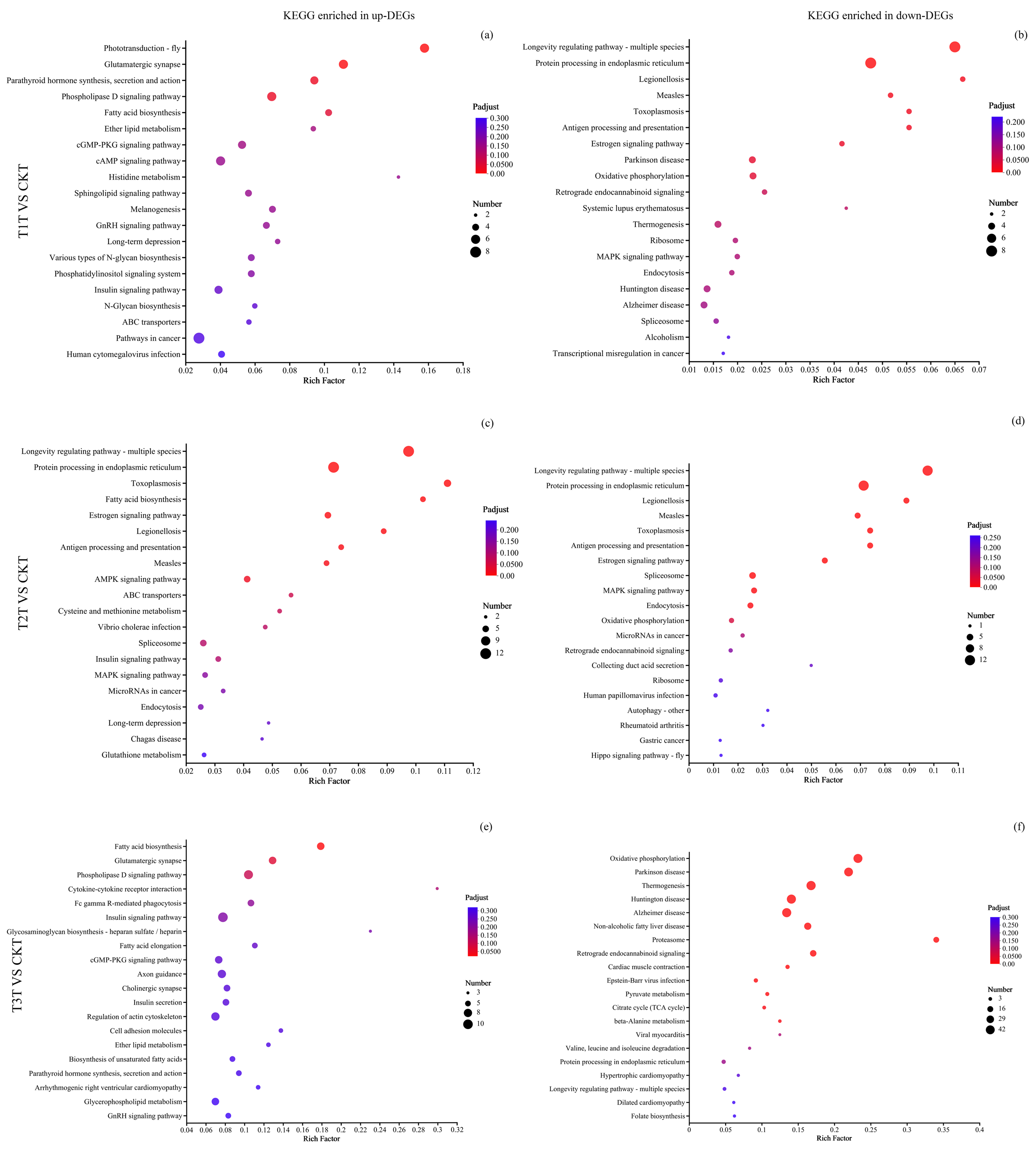

Supplement: Supplementary file 1 [file insects-15-00010-s001.zip › Fig. S7.tif]

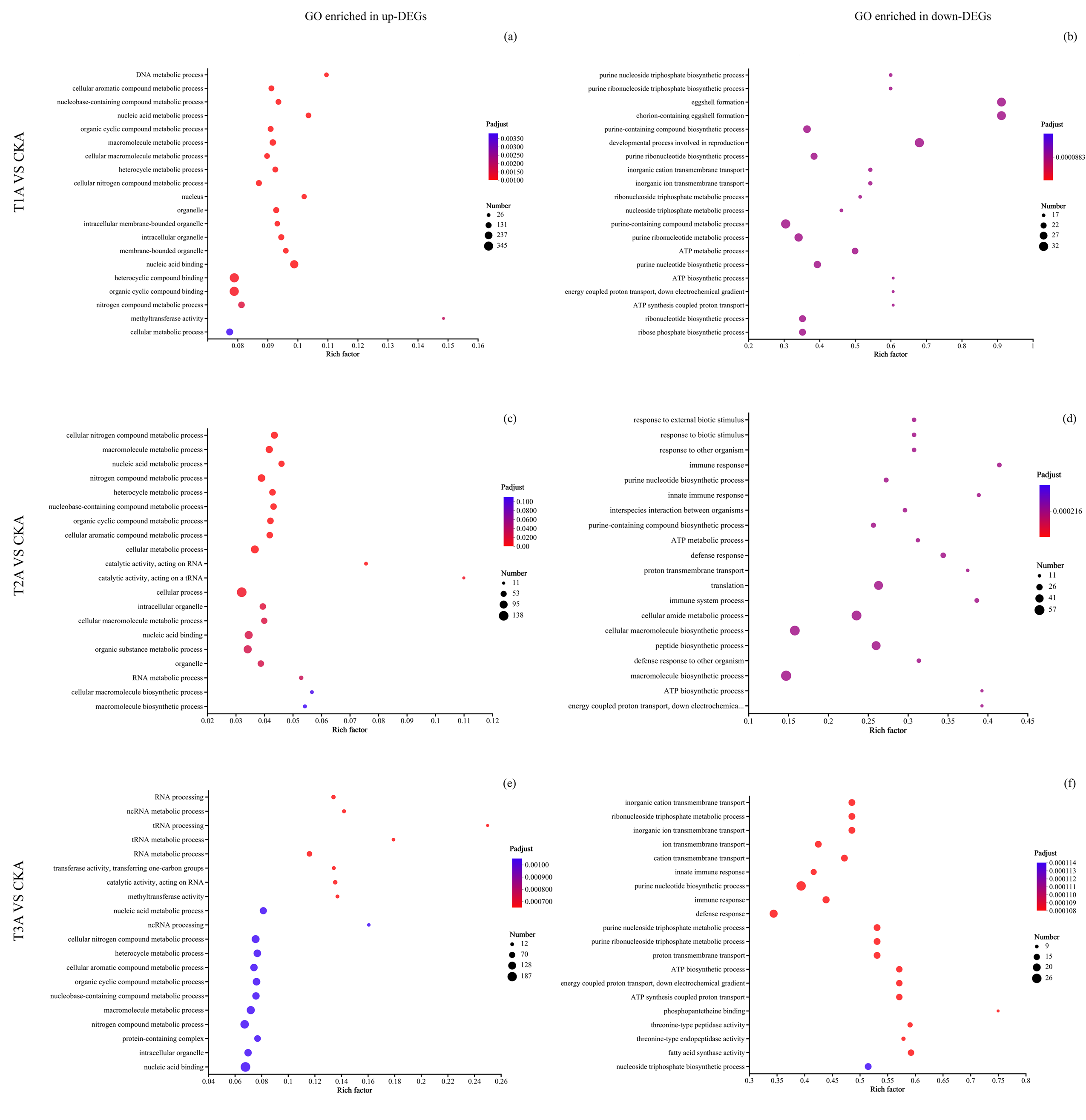

Supplement: Supplementary file 1 [file insects-15-00010-s001.zip › Fig. S8.tif]

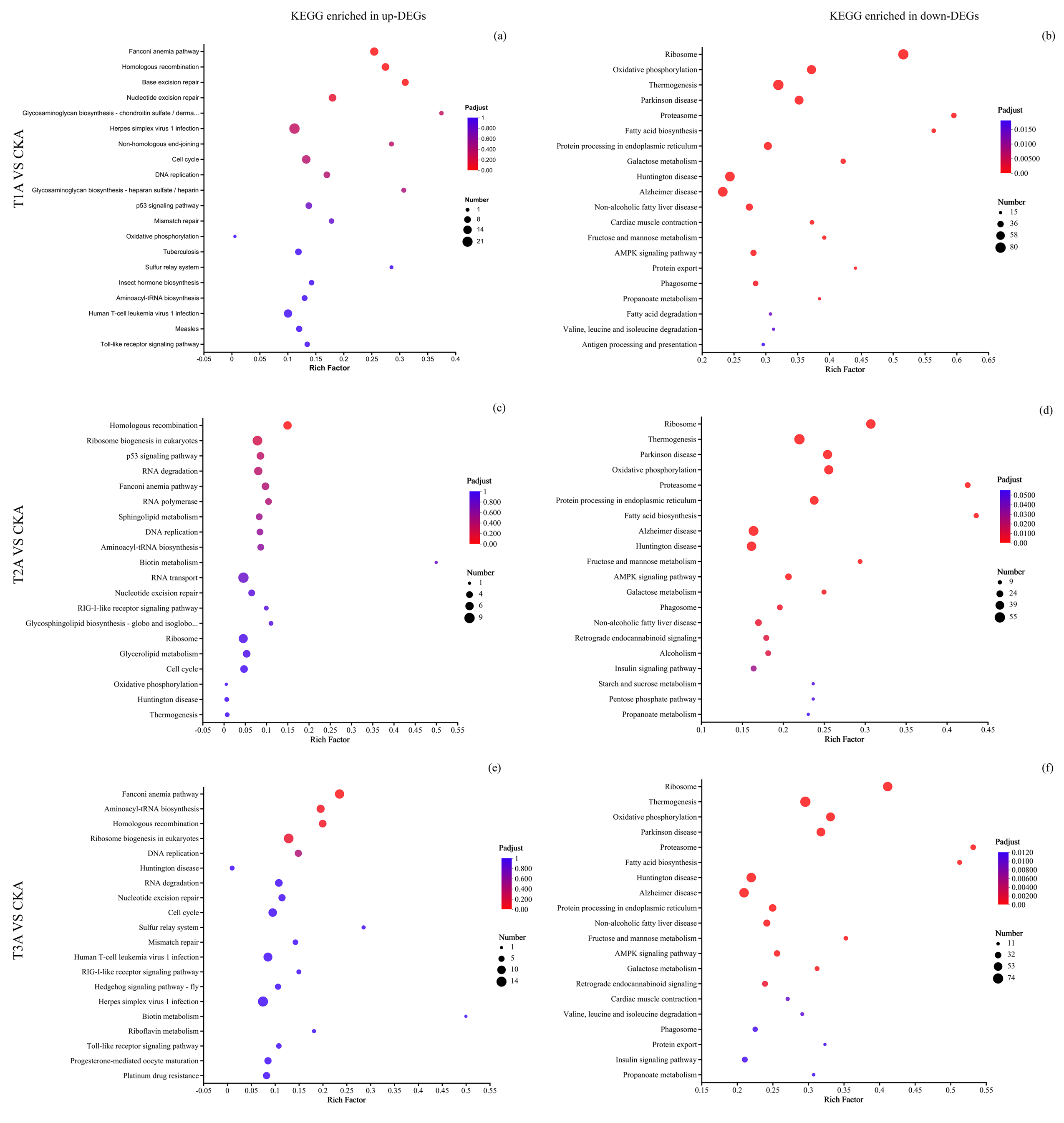

Supplement: Supplementary file 1 [file insects-15-00010-s001.zip › Fig. S9.tif]
